# Motor and somatosensory degenerative myelopathy responsive to pantothenic acid in piglets

**DOI:** 10.1177/03009858221128920

**Published:** 2022-10-17

**Authors:** Marina P. Lorenzett, Aníbal G. Armién, Luan C. Henker, Claiton I. Schwertz, Raquel A. S. Cruz, Welden Panziera, Claudio S. L. de Barros, David Driemeier, Saulo P. Pavarini

**Affiliations:** 1 Universidade Federal do Rio Grande do Sul, Porto Alegre, Brazil; 2 University of California, Davis, Davis, CA; 3 Universidade Federal de Mato Grosso do Sul, Campo Grande, Brazil

**Keywords:** ataxia, chromatolysis, conscious proprioception, cuneocerebellar tract, dorsal column-medial lemniscus pathway, motor neurons, pantothenic acid, paresis, spinocerebellar tract, swine, thoracic nucleus, unconscious proprioception, vitamin B5, Wallerian degeneration

## Abstract

This report describes 2 events of degenerative myelopathy in 4- to 27-day-old piglets, with mortality rates reaching 40%. Sows were fed rations containing low levels of pantothenic acid. Piglets presented with severe depression, weakness, ataxia, and paresis, which were more pronounced in the pelvic limbs. No significant gross lesions were observed. Histologically, there were degeneration and necrosis of neurons in the spinal cord, primarily in the thoracic nucleus in the thoracic and lumbar segments, and motor neurons in nucleus IX of the ventral horn in the cervical and lumbar intumescence. Minimal-to-moderate axonal and myelin degeneration was observed in the dorsal funiculus of the spinal cord and in the dorsal and ventral nerve roots. Immunohistochemistry demonstrated depletion of acetylcholine neurotransmitters in motor neurons and accumulation of neurofilaments in the perikaryon of neurons in the thoracic nucleus and motor neurons. Ultrastructurally, the thoracic nucleus neurons and motor neurons showed dissolution of Nissl granulation. The topographical distribution of the lesions indicates damage to the second-order neurons of the spinocerebellar tract, first-order axon cuneocerebellar tract, and dorsal column-medial lemniscus pathway as the cause of the conscious and unconscious proprioceptive deficit, and damage to the alpha motor neuron as the cause of the motor deficit. Clinical signs reversed and no new cases occurred after pantothenic acid levels were corrected in the ration, and piglets received parenteral administration of pantothenic acid. This study highlights the important and practical use of detailed neuropathological analysis to refine differential diagnosis.

Pantothenic acid (PA, vitamin B5) is a precursor for the biosynthesis of the phosphopantetheine moiety of coenzyme A (CoA) and acyl carrier protein.^21,36^ In the nervous system, CoA is central to the metabolism of neurons and glial cells. CoA is essential for diverse cellular metabolic processes, including the citric acid cycle, fatty acid biosynthesis, β-oxidation, cholesterol biosynthesis, and sphingolipid synthesis. Alterations in any of the steps of the CoA biosynthetic pathway can influence the proper functioning of any or all dependent processes.^20,36,41^ Acetyl-CoA, which is almost exclusively synthesized in the mitochondria in pyruvate dehydrogenase complex reaction, provides 97% of the neurons’ energy.^21,36^ Acetyl-CoA is particularly important for cholinergic neurons in the central nervous system (CNS) because they require additional amounts of acetyl-CoA for acetylcholine (Ach) synthesis in their cytoplasmic compartment to maintain their transmitter functions. Recent studies demonstrated that deficits in acetyl-CoA might be more harmful for cholinergic than for noncholinergic neurons in neurodegenerative diseases. The metabolism of CoA is also key for the cerebral biosynthesis of myelin. In contrast to other B-group vitamins, which are generally not stored in the brain, vitamin B5 is present throughout the brain at high concentrations and is localized largely within myelin of the white matter.^20,36,41^

PA is usually found in corn and soybean.^32^ Despite the natural bioavailability of this nutrient in these ingredients, PA is added to commercial diets fed to pigs of all ages and categories to mitigate the risk of deficiency.^10^ PA supplementation is usually performed through the addition of calcium pantothenate, a salt that is more stable than the compound itself and contains 46% of vitamin B5 in its active form.^25^

The onset of clinical signs of PA deficiency may be observed from approximately 7 to 10 days after the introduction of a vitamin B5–deficient diet.^7^ The disease is characterized by incoordination and ataxia affecting the fore and hind limbs.^16,34^ The main clinical manifestation of PA deficiency in pigs is described as the “goose-stepping gait,” which is characterized by gait changes, including hyperextension of the pelvic limbs associated with short steps.^7,16,34^

Case descriptions of PA deficiency are limited, probably because synthetic vitamins are supplemented.^10^ Nonetheless, the occurrence of outbreaks may be related to ration formulation errors, as well as operational issues involved in ration production, since in these cases a large number of animals may be affected. In addition, outbreaks may be associated with noncommercial diets in which PA is not added.^7^

Neurodegenerative disorders that more specifically or severely affect the spinal cord compared with the brain are relatively uncommon in swine, compared to other domestic animal species.^26^ Thus, a systematic examination of the CNS is necessary for the accurate diagnosis of degenerative disorders.^5,34^ A meticulous characterization of the nature of a lesion, anatomical localization, and affected functional system or unit is of great value in the diagnosis and comprehension of poorly understood degenerative, metabolic, or nutritional diseases.^9,34,39^ For instance, the identification of neurological systems by immunohistochemistry is of a greater significance, highlighting sensory axonal degeneration tracts aiming to diagnose equine degenerative myelopathy associated with vitamin E deficiency.^9^

Described herein is a neurodegenerative disorder with primary spinal cord involvement in suckling piglets, nourished from sows fed diets with deficient levels of PA. The first objective of the study was to describe the neuropathological, immunohistochemical, and ultrastructural findings of a PA-responsive myelopathy (PARM) in piglets. The second objective was to identify the functional neurological system or unit affected based on the topographical distribution of the lesions in the nervous system, providing new insights into the pathophysiology of this condition.

## Materials and Methods

### Clinical History and Epidemiology

Clinical and epidemiological information was obtained directly from field veterinarians and swine farm owners during the on-site visits. Twenty-two piglets of both sexes, aged between 6 hours and 27 days and presenting with neurological signs, were necropsied. In the first outbreak, pigs were referred from 2 farms (Farms 1 and 2) from the Santa Catarina state, Brazil. In the second outbreak, pigs were obtained from a single farm (Farm 3) located in the state of Goiás, Brazil.

The first outbreak occurred between June and July 2016. These 2 sow farms were associated with the same company, which provided a premix for the formulation of the ration fed to all pigs irrespective of the age categories. These farms reported increased mortality in piglets in the first week after farrowing and gait abnormalities affecting suckling, weaning, and growing-finishing pigs. Pigs in the affected farms presented with varied genetic makeup and were represented by several commercial breeds and crossbreeds. The onset of clinical signs was observed in suckling piglets, independent of age, from newborn piglets (first 6 hours after farrowing) to piglets aged 27 days of life. Most of the affected animals died within 48 hours of the onset of clinical signs. The total combined number of sows and gilts in each of the farms (Farms 1 and 2) ranged from 700 to 1000. Prior to the onset of the outbreak, the mortality rate of suckling piglets was approximately 5%. The outbreak lasted approximately 45 days in the 2 farms, and during this period, the mortality rate of suckling piglets ranged from 40% to 100%. Due to the likelihood of a nutrition-based disease, the premix company was contacted, which confirmed that to reduce production costs, vitamin B5 was removed from the premixes shortly before the outbreak occurred.

The second outbreak (Farm 3) occurred between June and July 2018 on a sow farm with wean-to-finish facilities. The farm had 1300 sows. During the outbreak, which lasted 60 days, the average mortality rate of suckling piglets increased from 6% to 40%. Some litters presented 100% mortality rate up to the fifth day after farrowing. Clinical signs were observed in suckling piglets as young as 6 hours of age and were seen in piglets of all ages throughout the phase until weaning (27 days of life). The litters of gilts and multiparous sows were affected. Approximately 15% of the suckling piglets started presenting gait abnormalities at weaning, which were similar to those observed during the first outbreak. In this outbreak, it was investigated and determined by the referral company that PA-deficient levels in lactation and gestation premixes were due to a formulation error associated with a failure in an operational process. The clinical manifestations in the piglets were documented through detailed history, observation of the animals, sequential photographs, and videos. Neurological signs were evaluated using parameters defined previously.^2,5^ As the examination of reflexes, postural reactions, touch, and pain abnormalities were not performed, this information is not available for this study. The gathered neurological information was analyzed in conjunction with the topographical distribution of the lesions in the CNS and peripheral nervous system (PNS). Neurological signs were classified according to their severity and subjectively graded as mild, moderate, or severe. Mild neurological signs were usually not well defined with intermittent periods of manifestation, and piglets presented with depression, weakness, and a low level of difficulty in performing coordinated voluntary movements. Moderate neurological signs were well defined with intermittent to prolonged periods of manifestation, and the piglets exhibited an intermediary level of difficulty in performing coordinated voluntary movements. Severe neurological signs were well to poorly defined, and the piglets displayed sustained and high-level difficulty in performing coordinated voluntary movements to loss of function (Table 1).

**Table 1.** Neurological deficits and topographical distribution of lesions affecting motor and somatosensory nuclei and pathways of the spinal cord in piglets with pantothenic acid–responsive myelopathy.

Pig ID	Age (Days)	Locomotion Deficits		Spinal Cord									
				Motor System			Somatosensory System						
		Front Limbs	Hind Limbs	Nuclei IX			Thoracic Nucleus		Dorsal Funiculus			Lateral Funiculus^a^	
				Cervical	Thoracic	Lumbar	Thoracic	Lumbar	Cervical	Thoracic	Lumbar	Cervical	Thoracic
1	5	−^b^	+	−	−	−	(+)	−	−	−	−	−	−
2	6	−	++	−	−	−	(+)	−	−	−	−	−	−
3	7	+	++	−	−	+(+)	−	+	−	−	−	−	−
4	15	++	++	−	−	−	−	+	−	−	−	−	−
5	10	++	++	−	(+)	+(+)	+(+)	+	−	−	−	−	−
6	11	++	+++	(+)	−	++	−	+	+(+)	−	+(+)	−	−
7	8	+	++	(+)	−	+	−	+	−	−	(+)		−
8	15	+++	+++	+(+)	+	−	+	++	+(+)	+(+)	+(+)	(+)	(+)
9	4	++	+	−	−	−	(+)	Not analyzed	(+)	(+)	(+)	(+)	(+)
10	5	+	−	(+)	(+)	+	−	−	+	+	+(+)	(+)	(+)
11	20	+	+++	(+)	−	(+)	+	+++	−	−	−	−	−
12	21	++	+++	(+)	+	−	+	++	−	−	−	−	−
13	4	+	++	(+)	−	−	+	++	−	−	−	−	−
14	5	+	+	−	−	−	(+)	(+)	−	−	−	−	−
15	11	+++	+++	(+)	(+)	+	+(+)	+++	(+)	(+)	+	(+)	(+)
16	27	+++	+++	−	−	+	+	++(+)	(+)	++	++	(+)	(+)

a Spinocerebellar tract.

b +++ = severe; ++ = moderate; + = mild; (+) = discreet; − = no lesion.

Piglets were euthanized, and necropsy was performed on the farms. The study was approved by the Veterinary Research Commission of the Federal University of Rio Grande do Sul (Approval No. 33526).

### Postmortem Examination

Necropsies were performed on 22 affected and 2 control piglets. Brain, spinal cord, peripheral nerves, heart, lungs, kidneys, urinary bladder, liver, pancreas, esophagus, stomach, small and large intestines, thyroid, adrenal glands, tonsils, spleen, mediastinal and mesenteric lymph nodes, and skeletal muscle were collected and fixed in 10% buffered formalin. The tissues were embedded in paraffin, sectioned, and stained with hematoxylin and eosin. Additional representative sections of the spinal cord were stained with Bielschowsky.

### Neuropathology

The skull, vertebral column, encephalon, spinal cord, and peripheral nerves were carefully examined for any gross abnormalities. To determine the nature and distribution of the lesions in the CNS and PNS, tissue sections of the diseased animals were screened for inflammatory, circulatory, and degenerative changes.^22,34^ The examined areas of the CNS encompassed the telencephalon (coronal section at 3 levels), basal nuclei (coronal section at 1 level), thalamus (coronal section at 1 level), cerebellum (horizontal section at 1 level), mesencephalon (coronal section at 1 level), and pons and medulla oblongata (coronal sections at 2 levels). Cross-sections of the spinal cord were examined in the following regions/segments: cervical (C1, C2, C5–C7), thoracic (T2, T10, T13), lumbar (L1, L3, L4–L6), sacral (3 levels), and coccygeal (1 level). All the dorsal and ventral nerve roots of the lumbosacral segments and dorsal root ganglia were examined. In addition, transverse and longitudinal sections of the sciatic nerve, brachial plexus, semimembranosus, semitendinosus, and gastrocnemius were examined.

Having determined that the primary pathological process was degenerative in nature, a systematic evaluation of the neural centers and pathways associated with somatosensory and motor control was performed.^2,5,9,22,34,39^ Two age-matched pigs were used as controls. For each spinal cord segment (cervical, thoracic, and lumbar), 2 to 6 histological sections were examined. CNS tissue sections were evaluated by 2 authors (M.P.L. and A.G.A.).

Qualitative and semiquantitative neuronal degeneration and/or necrosis, and axonal and myelin degeneration were assessed in the following areas:

- Cerebrum, including the gyri sigmoideus, marginalis, ecto marginalis, suprasylvius, ectosylvius, and cinguli.
- Nucleus basalis, caudatus, and putamen;
- Thalamus, globus pallidus, capsula interna, externa, extrema, and ventral thalamic nuclei;
- Mesencephalon: tectum, nucleus ruber, substantia nigra, oculomotor nuclei, and formatio reticularis;
- Cerebellum: folia of the cerebellar vermis and hemispheres, nucleus lateralis, interpositus, fastigii, and vestibular.
- Brainstem: nuclei cuneatus lateralis, gracilis, olivae, vestibularis, and formatio reticularis;
- Gray and white matter of the cervical, thoracic, lumbar, sacral, and coccygeal spinal cord.

Histological changes in the spinal cord were graded according to the severity level: minimum (+), mild +, mild/moderate +(+), moderate ++, moderate/severe ++(+), and severe +++. This initial qualitative and semiquantitative screening of the CNS and PNS permitted identification of the primarily affected areas of the spinal cord. Subsequently, due to the variability of the neuronal population in individual cervical, thoracic, lumbar, and sacral spinal cord segments, a semiquantitative evaluation specifically assessing nucleus IX of the ventral horn and thoracic nucleus was performed. The scale developed in this study considers the average number of degenerate neurons viewed in both the right and left thoracic nuclei, and nucleus IX of the ventral horn. In the ventral horns of the spinal cord, an average of 30 neurons were evaluated on each side. In the thoracic nucleus in control and in diseased piglets, the average number of neurons observed on each side (right and left) was 11. Based on the number of degenerated neurons, the lesion scale was determined as follows: lesions in nucleus IX of the ventral horn were considered minimal when up to 3 were affected, mild when 4–5 were found, mild to moderate when 6–7 were detected, moderate when 8–10 were observed, moderate to severe when 11–14 were found, and severe when 15 or more were involved. In the thoracic nucleus, lesions were considered minimal when 1 degenerated neuron was detected, mild when 2–3 were observed, mild to moderate when 4–5 were found, moderate when 6–7 were detected, moderate to severe when 8–10 were present, and severe when 11 or more neurons were identified. Axonal and myelin degeneration scales were set according to the number of axonal spheroids and digestion chambers observed in the nerve roots and white matter of the spinal cord. Minimal axonal lesions were <5, mild lesions were 6–8, mild to moderate lesions were 9–11, moderate lesions were 12–14, moderate to severe lesions were 15–17, and severe lesions were > 20. Six of the 22 piglets with neurological signs that were subjected to necropsy did not show any histological changes. The presumed causes of death in these animals include hypoglycemia, dehydration, hypothermia, or crushing. Thus, these 6 piglets were not included in the study because of the lack of validated tests to detect and measure the levels of PA deficiency in animal tissues.

### Immunohistochemistry

Immunohistochemical staining using monoclonal and polyclonal antibodies was performed on representative sections from the thoracic and lumbar spinal cord of 2 severely affected piglets (details are provided in Supplemental Table S1). Immunohistochemistry of the neuronal cytoskeletal proteins, non-phosphorylated and phosphorylated neurofilaments (NFs), calcium-binding protein involved in neuronal calcium signaling, calretinin, and the neuromuscular junction neurotransmitter enzyme choline acetyltransferase was undertaken. In addition, to determine the astroglial and microglial responses, immunostaining for glial fibrillary acidic protein and microglial ionized calcium-binding adaptor molecule 1 was performed. Immunohistochemistry was performed using an automated slide stainer (Dako, Carpinteria, CA, USA), and a peroxidase-labeled polymer conjugate system (Dako) was used as a secondary antibody. Sections of 4 µm thickness were deparaffinized and rehydrated in a graded alcohol series. Antigens were unmasked by the heat-induced epitope retrieval method using a Biocare Decloaking Chamber (Biocare Medical, Concord, CA, USA) and a retrieval buffer of pH 6.0 or 9.0. Endogenous peroxidase was blocked with 3% hydrogen peroxide for 15 minutes. Nonspecific binding sites were blocked with normal goat serum (1:10 in Tris-buffered saline) for 15 minutes. The slides were then incubated with the primary antibody. Thereafter, the sections were incubated with a horseradish peroxidase–conjugated secondary antibody. Immunoreactivity was detected using 3-amino-9-ethylcarbazole+ for 5 to 15 minutes. Slides were lightly counterstained with Mayer’s hematoxylin for 5 minutes.^9^ For detection of ionized calcium binding adaptor molecule 1, heat-induced antigen retrieval was performed prior to incubation with primary antibodies in a premade buffer (Biocare).^38^

Swine spinal cord was used as a positive control for anti-phosphorylated NF, anti-non-phosphorylated NF, anti-calretinin, anti-choline acetyltransferase, and anti-GFAP. Swine lymph node was used as a positive control for anti-Iba-1. Negative control included the primary antibody replaced by either homologous nonimmune sera or an isotype-matched nonrelevant antibody.

### Electron Microscopy

For electron microscopy evaluation, brain, spinal cord, and spinal ganglia fragments of 2 piglets from Farm 3 were fixed with 2% glutaraldehyde. Fragments of 1 to 3 mm were postfixed in 2.5% glutaraldehyde (Electron Microscopy Sciences, Hatfield, PA, USA) in 0.1 M sodium cacodylate buffer (Electron Microscopy Sciences). Tissue samples were postfixed in 1% osmium tetroxide (Electron Microscopy Sciences) in 0.1 M sodium cacodylate buffer, dehydrated, and embedded in resin as previously described.^39^ Thin sections (60–70 nm) were stained with 5% uranyl acetate and lead citrate. The samples were visualized using a JEOL 1400 Plus transmission electron microscope (JEOL LTD, Tokyo, Japan). Images were obtained using an AMT Capture Engine Version 7.00 camera and software (Advanced Microscopy Techniques Corp., Woburn, MA, USA) and analyzed using ImageJ software (National Institute for Health and Care Research [NIHR] Public Domain).

### Determination of PA Concentration

Samples of lactation premix and ration (Farm 1) from outbreak 1 were collected. Premix and rations of gestation and lactation phases from outbreak 2 (Farm 3) were also sampled to assess and measure calcium pantothenate. Samples from outbreaks 1 and 2 were submitted to CBO Analysis Laboratory in São Paulo, Brazil. High-performance liquid chromatography was used to measure the vitamin B5 levels. The limit of detection of vitamin B5 using this technique is 5.0 mg/kg, and the limit of quantification is 7.0 mg/kg.^19^ Tests to determine PA in tissues were not available.

## Results

**Figure 1.**
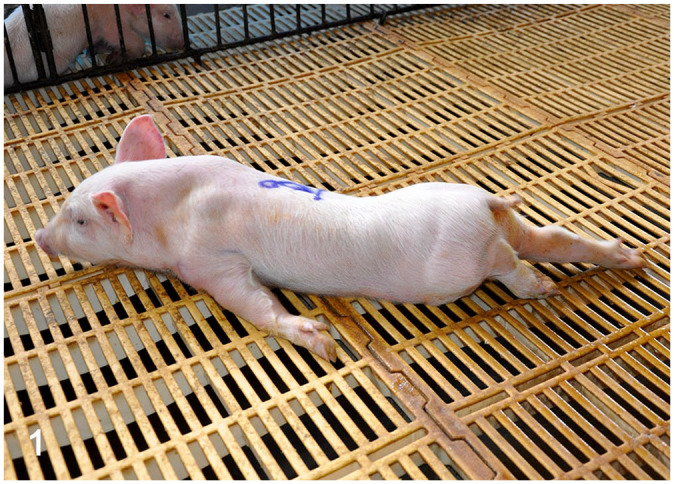
Pantothenic acid–responsive degenerative myelopathy, Pig 16. Animal with tetraparesis. The ventral abdominal surface is in contact with the floor, and the forelimbs are flexed or laterally positioned. The head and ears, however, maintain a normal orientation.

The clinical manifestations were mainly characterized by locomotion deficits that occasionally evolved to tetraparesis (Fig. 1 and Supplemental Videos S1–S3). Locomotion deficits were more severe in pelvic limbs. However, there was large variation between the front and posterior limbs and among animals (Table 1). The clinical signs in the first week after birth were dominated by severe depression and weakness (paresis). Piglets showed dropped head and neck, knuckling over, hind limb hypermetry, and prolonged periods in sternal recumbence with splayed limbs or limbs in forward and backward positions (paraparesis and tetraparesis). Piglets in their second week of life exhibited prominent clinical signs. Animals presented with knuckling, which was observed in all 4 limbs in most animals, and exhibited severe paresis. Piglets supported their body weight in the tarsus-metatarsus region, presenting light to marked “hock weight-bearing” and “hock walking” (Supplemental Fig. S1). Furthermore, piglets showed “goose-stepping gait.” Animals also developed hypermetry, instability, and incoordination, which commonly led to falls (sensory ataxia; Supplemental Videos S1, S2). Some animals demonstrated abnormal and irregular alternation of movements (dysdiadochokinesia). Piglets with a longer clinical course were unable to stand up and support weight, adopting a dog sitting position or sternal recumbence with splayed limbs or limbs in the forward and backward positions (paraparesis and tetraparesis). Some piglets performed continuous movements of all 4 limbs in an attempt to walk as if they were swimming in a pool (tetraparesis; Supplemental Video S3). Other clinical signs included anorexia, depression, drowsiness, and diarrhea, which was unresponsive to antibiotic therapy in piglets in the first week.

At necropsy, gross findings were nonspecific and included a yellow liver (fatty degeneration) and watery contents in the intestinal lumen.

The most important microscopic findings were observed in the spinal cord (Tables 1 and 2). Microscopic lesions were observed in 16 (72.73%) of 22 piglets and varied from discrete to severe. In addition, scattered necrotic neurons (ranging from 1 to 8 neurons) were observed in the reticular formation of the brainstem in 4 of 16 piglets. No microscopic changes were observed in the remaining portions of the brain cortex, basal nuclei, thalamus, mesencephalon, cerebellum, spinal ganglion, brachial plexus, sciatic nerves, or muscles.

**Table 2.** Distribution of the axonal and myelin degeneration affecting somatosensory pathways of the spinal cord in piglets with pantothenic acid–responsive myelopathy.

Piglet No.	Somatosensory Pathway													
	Cervical					Thoracic					Lumbar		Sacral	
	Dorsal Funiculus			Lateral Funiculus		Dorsal Funiculus			Lateral Funiculus		Dorsal Funiculus	Lateral Funiculus	Dorsal Funiculus	Lateral Funiculus
	FP	FG	FC	DSCT	VSCT	FP	FG	FC	DSCT	VSCT				
6	+(+)	(+)	+	(+)	(+)	(+)	(+)	(+)	(+)	(+)	(+)	(+)	−	−
7	(+)	(+)	(+)	(+)	(+)	(+)	(+)	(+)	(+)	(+)	−	−	−	−
8	+(+)^a^	(+)	+(+)	(+)	(+)	+	(+)	++	(+)	(+)	−	−	−	−
9	(+)	(+)	(+)	(+)	(+)	+	(+)	+	(+)	(+)	−	−	−	−
10	+	+	+(+)	(+)	(+)	(+)	(+)	+(+)	(+)	(+)	+(+)	(+)	(+)	(+)
15	(+)	(+)	(+)	(+)	(+)	(+)	(+)	(+)	(+)	(+)	−	−	−	−
16	(+)	(+)	(+)	(+)	(+)	(+)	(+)	+	(+)	(+)	+(+)	(+)	(+)	(+)

Abbreviations: FP, fasciculus proprius; FG, fasciculus gracilis; FC, fasciculus cuneatus; SCT, spinocerebellar tract; DSCT, dorsal spinocerebellar tract; VSCT, ventral spinocerebellar tract.

a +++ = severe; − = no lesion; ++ (+) = moderate/severe; ++ = moderate; + (+) = mild/moderate; + = mild; (+) = minimum.

**Figures 2–7.**
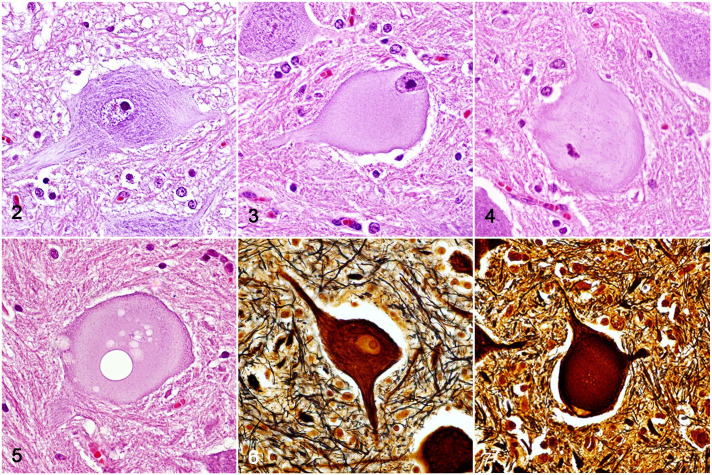
Pantothenic acid–responsive degenerative myelopathy, spinal cord, pig, cases 15 and 16. **Figure 2.** Motor neuron, morphologically normal. Hematoxylin and eosin (HE). **Figure 3.** Degenerate neurons displaying swollen, pale, homogeneous, and eosinophilic cytoplasm. Nissl granules are dissolved in the center of the cell body, and the remaining granules are in the periphery of the neuronal soma. The nucleus is peripherally displaced (HE). **Figure 4.** Necrotic neuron, eosinophilic with nucleus showing pyknosis (HE). **Figure 5.** Necrotic neuron with multiple vacuoles (HE). **Figure 6.** Normal (control) neuronal body. Bielschowsky. **Figure 7.** Necrotic neurons swollen with neurofibril accumulation predominantly on the periphery of the soma. Bielschowsky.

**Figures 8–10.**
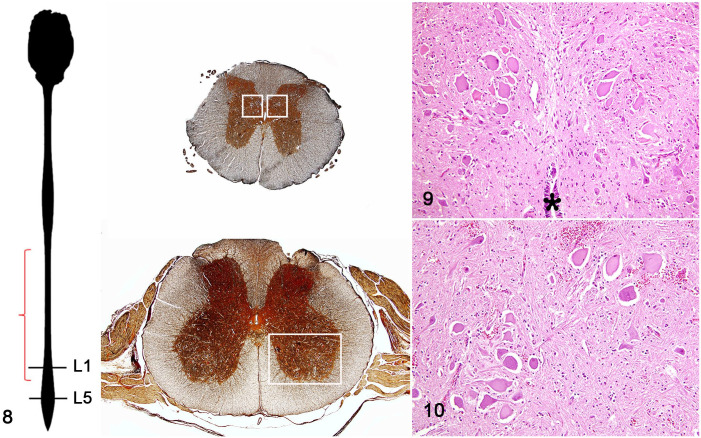
Pantothenic acid–responsive degenerative myelopathy, spinal cord, pig, case 15. **Figure 8.** Left, a diagram of the central nervous system (CNS) depicting the location of the thoracic nucleus, which extends from the thoracic vertebra T1 to the lumbar vertebra L3 (red bracket). Right top image shows L1 transverse section; location of thoracic nuclei (white rectangles). Right bottom image shows L5 transverse section; location of lower motor neuron nuclei in the ventral horns (white rectangle). **Figure 9.** Necrosis and degeneration of sensory neurons located in the thoracic nucleus in the L1 lumbar segment (hematoxylin and eosin [HE]). *Central spinal cord canal. **Figure 10.** Necrosis and degeneration of motor neurons of nucleus IX located in the ventral horns of the spinal cord in the L5 lumbar segment (HE).

The lesions in the spinal cord were characterized by mild to severe neuronal degeneration, necrosis, and axonal and myelin degeneration. Degenerated neurons were swollen with pale and eosinophilic cytoplasm, in part due to loss of Nissl bodies, which was more evident in the central perikaryon (chromatolysis). The nucleus in several of these neurons was peripherally displaced (Figs. 2, 3). Pyknosis or the absence of the nucleus indicated unequivocal neuronal cell death (Fig. 4). Some necrotic neurons were shrunken, hypereosinophilic, and lacked nuclei. Some necrotic cells were surrounded by microglia, as confirmed by immunohistochemistry (Supplemental Fig. S3), with occasional neuronophagia (image not shown). A few necrotic neurons were vacuolated (Fig. 5). On Bielschowsky histochemical staining, degenerated and necrotic neurons were swollen, with disintegration of the “neurofibrils,” which remained accumulated in the periphery of the soma (Figs. 6, 7). Degenerated and necrotic neurons were found primarily in the thoracic nucleus at the base of the dorsal horns and in nucleus IX in the ventral horns of the spinal cord (Figs. 8–10, Table 1).

**Figures 11–14.**
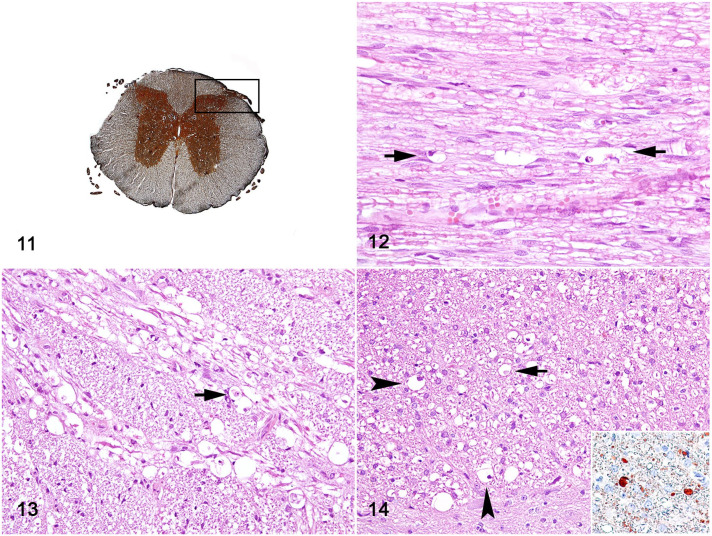
Pantothenic acid–responsive degenerative myelopathy, spinal cord, pig, case 15. **Figure 11.** L1 spinal cord segment; the box delimits the dorsal nerve root, dorsal root entry zone, and dorsal funiculus. Bielschowsky. **Figure 12.** Dorsal nerve root, between arrows, a distended myelin sheath containing a presumptive gitter cell (macrophage) is seen. **Figure 13.** Dorsal root entry zone, distended myelin sheath containing presumptive gitter cells (arrow). **Figure 14.** Dorsal funiculus displaying distended myelin sheath with swollen axon (arrow) and presumptive gitter cells (arrowheads). Hematoxylin and eosin. Inset: Immunohistochemistry for calretinin (brown labeling) highlighting axonal spheroids.

In addition, white matter lesions, which were characterized by variable degrees of axonal and myelin degeneration with macrophage infiltration (gitter cells), were observed in the dorsal and ventral roots, and the root entry zone of the spinal cord. Axonal spheroids, along with myelin degeneration and gitter cells, were more prevalent in the dorsal funiculus, especially in the thoracic and lumbar spinal cord segments (Figs. 11–14); however, these changes were not consistently observed in all sections.

Although lesions in the spinal cord were bilateral, they were asymmetrical. The number of degenerated and necrotic neurons detected in hematoxylin and eosin sections varied among spinal cord segments, as shown in Table 1. Degeneration or necrosis of neurons in nucleus IX and in thoracic nucleus was observed in 11 (68.75%) of 16 piglets and in 15 (93.75%) of 16 piglets, respectively. Axonal and myelin degeneration in the dorsal and lateral funiculi were observed in 7 (43.75%) of 16 piglets. Further analysis of these funiculi showed that all 3, proper, gracilis, and cuneate fasciculi, and both dorsal and ventral spinocerebellar tracts were affected (Table 2).

**Figures 15–18.**
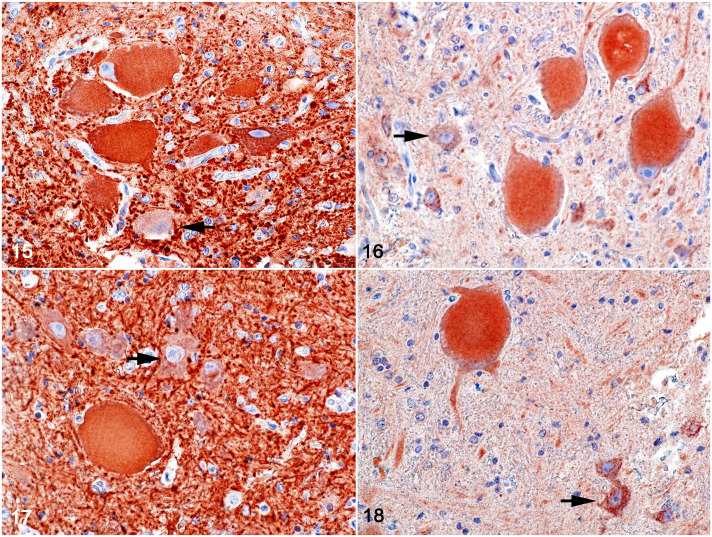
Pantothenic acid–responsive degenerative myelopathy, L1 spinal cord segment, pig, case 15. Immunohistochemistry for phosphorylated and non-phosphorylated neurofilament (brown labeling). **Figure 15.** Necrotic neurons of the thoracic nucleus displaying accumulation of phosphorylated neurofilament within the cell bodies as well as within cell processes; an unaffected neuron (black arrow). **Figure 16.** Necrotic neurons of the thoracic nucleus demonstrating accumulation of non-phosphorylated neurofilament within cell bodies; an unaffected neuron (black arrow). **Figure 17.** Necrotic motor neurons in nucleus IX displaying accumulation of phosphorylated neurofilament within the cell bodies as well as within cell processes; an unaffected neuron (black arrow). **Figure 18.** Necrotic neurons of the motor nucleus IX displaying accumulation of non-phosphorylated neurofilament within the cell bodies; an unaffected neuron (black arrow).

**Figures 19–21.**
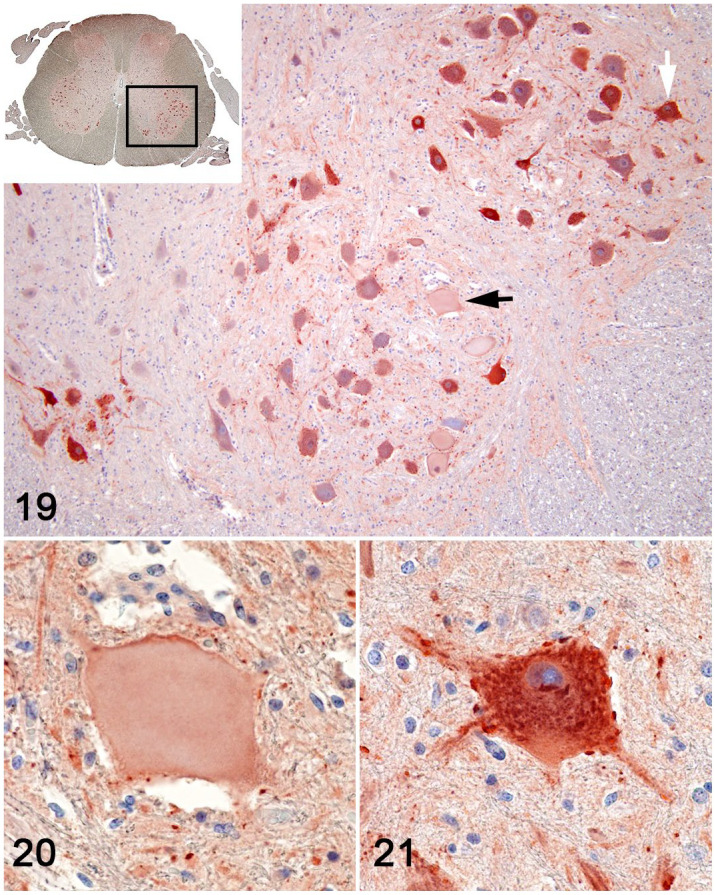
Pantothenic acid–responsive degenerative myelopathy, L5 spinal cord segment, pig, case 15. Choline acetyltransferase immunohistochemistry (brown labeling). **Figure 19.** Degeneration and necrosis of motor neurons in the nuclei, in the ventral horns of L5 (magnification from the box in the Inset); an affected neuron (black arrow); an unaffected neuron (white arrow). **Figure 20.** Magnified degenerated/necrotic motor neuron from Fig. 19, demonstrating marked depletion of choline acetyltransferase. **Figure 21.** Unaffected motor neuron. While the degenerate/necrotic motor neurons displayed light gray-brown homogeneous cytoplasm, unaffected neurons showed abundant course brown granulation.

Axonal spheroids in the roots of the dorsal and ventral nerves and dorsal funiculus were diffusely immunoreactive for calretinin (Fig. 14 inset). Compared with unaffected neurons, an accumulation of phosphorylated and non-phosphorylated NFs in necrotic neurons of nucleus IX (Figs. 15, 16) and thoracic nucleus (Figs. 17, 18) was detected by immunohistochemistry. As demonstrated by choline acetyltransferase immunohistochemistry, low expression of the ACh neurotransmitter was observed in the motor neurons (MNs) of nuclei IX. The degenerated MN had a clear cytoplasm with no granulations compared with the unaffected neurons (Figs. 19–21). The presence of ionized calcium-binding adaptor molecule 1 highlighted that the predominant cell population of the glial cell response was represented by microglial cells (Supplemental Fig. S3).

**Figures 22–25.**
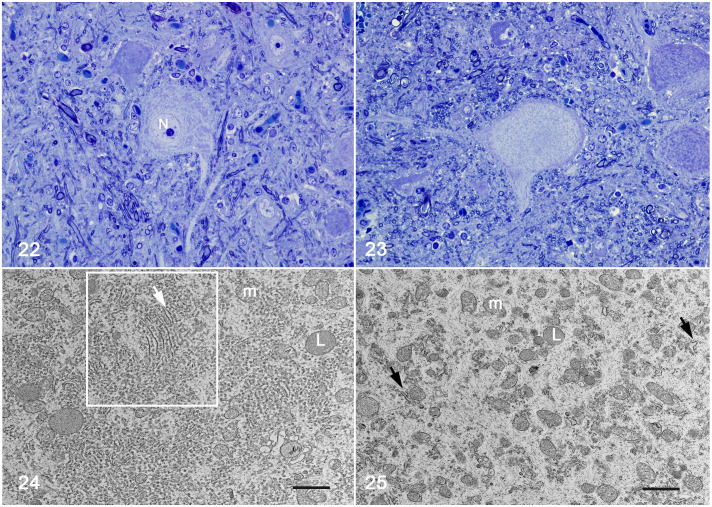
Pantothenic acid–responsive degenerative myelopathy, L5 spinal cord segment, pig, case 15. **Figure 22.** Unaffected neuron; nucleus (N). **Figure 23.** Affected ventral horn motor neuron presenting dissolution of the Nissl bodies and absence of the nucleus. Toluidine blue. **Figure 24.** Ultraphotomicrophotograph of neuron in Fig. 22, depicting unaffected Nissl substance. The white border of the square delineates aggregates of rough endoplasmic reticulum with parallel cisterns (white arrow) surrounded by abundant polyribosomes, mitochondria (M), and lysosomes (L). **Figure 25.** Ultraphotomicrophotograph of neuron in Fig. 23, demonstrating degranulation of rough endoplasmic reticulum and disaggregation of polyribosomes. Few small cisterns of endoplasmic reticulum (black arrows) appear along with a relatively increased number of mitochondria (M), increased intermediary filaments, and few lysosomes (L). Transmission electron microscopy. Scale bar= 1 µm.

Ultrastructural changes were detected in the lumbar segment of the spinal cord in 1 of the 2 animals (piglet 15). Neurons of the thoracic nucleus and MN showed dissolution of the Nissl substance with a redistribution of organelles (Figs. 22–25). In the center of the neuron cell body, there was a paucity of endoplasmic reticulum cisterna and polyribosomes that compose the Nissl substance. Amid scant organelles, mostly mitochondria, were the sparsely distributed intermediary filaments (Figs. 24, 25). The remaining rough endoplasmic reticulum aggregates were displaced to the soma periphery (Figs. 24, 25). Degenerate axons were sparsely distributed and characterized by the accumulation of residual bodies, mitochondria, and vesicles among sparse intermediary filaments. Frequently, these axons were surrounded by a thin myelin sheath exhibiting segmental decompaction. In the center of the degenerate myelinated axonal tube, macrophages often phagocytize axons and myelin debris (digestion chamber, Supplemental Figs. S4, S5). No significant ultrastructural changes were observed in the spinal ganglia or the nerves.

The assessment of calcium pantothenate concentration showed that the levels in the lactation ration samples (outbreak 1) were lower than 7 mg/kg, while in the lactation premix samples the concentration was 35.73 mg/kg. The analysis of premix and ration samples of gestation and lactation phases (outbreak 2) indicated calcium pantothenate levels were lower than 7 mg/kg, which made quantification not feasible.

After the deficiency (lack or low concentration levels) of dietary PA was identified in the rations fed to the affected piglets, injectable and oral (drinking water) vitamin supplementation was administered using commercial preparations containing vitamin B5. In addition, calcium pantothenate was added to the ration. After rectifying for PA dietary deficiency, a marked reduction in neonatal mortality was noted. Furthermore, the observation of new cases of proprioceptive deficits ceased within 10 days of the supplementation.

## Discussion

In this study, we describe an outbreak of a neurological condition in suckling piglets, characterized by degenerative myelopathy. The distribution and severity of the degenerative changes in spinal cord neurons and tracts, and dorsal and ventral spinal nerve roots of affected piglets were consistent with proprioceptive and motor deficits.^2,5^ Common circumstances between the 2 outbreaks were: (1) deficient levels of PA in ration fed to pregnant and lactating sows due to ration formulation errors, and (2) after adjusting the dietary requirements of PA in the ration fed to swine, piglets presenting proprioceptive and motor deficits recovered and the mortality ceased.

PA deficiency has been reported in pigs fed diets based exclusively on corn and diets based on self-made rations with human food leftovers comprising fish, meat, and bone. In both situations, PA was not supplemented.^33,34^ In the current outbreaks, PA deficiency has been noted in farms in which corn and soybean are the main ration ingredients, showing that the bioavailability of naturally occurring vitamin is insufficient to meet the metabolic demands and prevent clinical symptoms.^10^ Suckling piglets were the most affected age category. This can be attributed to the fact that gestating sows were having PA-deficient diets for over half of their gestation period because of errors in the ration formulation. Furthermore, sows on deficient diets nursed piglets during the lactation period when pigs had a high demand for PA. In swine, the highest requirement for PA is presented in piglets up to 15 kg of body weight.^17^ The PA requirement of piglets weighing 2 to 10 kg is 15.0 mg/kg of body weight.^25^ This requirement is significantly higher than the levels detected in the rations fed (below 7 mg/kg) to gestating and lactating sows in the reported outbreaks. However, a lower level of PA is expected for the newborn piglet due to losses associated with intestinal absorption as they also developed diarrhea. After adjusting the dietary levels of PA in the ration fed to swine of all age categories in the affected farms, clinical cases related to PA deficiency were reversed, and no new cases were reported. In addition to dietary supplementation, PA was added to drinking water. Piglets presenting with clinical signs were also injected intramuscularly with vitamin B5. After parenteral therapy and dietary supplementation, an immediate reduction in neonatal mortality was observed. The response of clinically diseased pigs to daily calcium pantothenate supplementation has also been reported elsewhere. For example, a study reported that pigs presented rapid and complete recovery and a great improvement in feed conversion following the supplementation.^16,40^ The clinical disease observed in suckling piglets likely favored increased mortality rates associated with secondary causes of noninfectious origin, including severe dehydration, increased piglet crushing rates, hypothermia, and hypoglycemia, all of which represent critical survival and viability parameters for newborn piglets in swine production systems.^37^ In the present study, the lower viability and reduced birth weight of piglets may be related to inadequate energetic intake and consequent ineffective cellular metabolism associated with CoA deficiency in the gestation period.^22^ Similarly, the causes of death of the 6 piglets that were excluded from the study could be also associated with the rapid progression of clinical signs leading to death due to secondary causes before the development of detectable microscopic lesions arising due to the deficiency of PA.

A major limitation of our study was the inability to determine the levels of vitamin B5 in tissues and the consequent lack of reference values for tissue measurements of PA in swine. Furthermore, α-tocopherol (vitamin E) and tissue mineral levels such as selenium and copper were not measured. Moreover, viral diseases were not investigated in this study. The epidemiological clinical data and the absence of characteristic microscopic lesions, however, rule out the aforementioned conditions as potential causal factors toward differential diagnoses, as further discussed.

The exact mechanism of neuronal cell damage caused by PA deficiency in pigs remains unclear. However, it is recognized that the absence or low level of vitamin B5 interferes directly with the synthesis of ubiquitous CoA, which is required for energy production in the mitochondria and diverse synthetic pathways in various extra-mitochondrial compartments. Thus, excessive energy depletion is the most likely cause of neuronal cell damage and death, especially in cholinergic neurons, which have a higher demand for acetyl-CoA to maintain their transmitter functions.^20,34,36,41^ Neuronal cell death due to excessive energy depletion includes several causal modalities such as ischemia, hypoglycemia, excitotoxicity, nutritional deficiencies, and exposure to toxins and may involve a series of complex mechanisms with distinct morphological features such as necrosis, apoptosis, and autophagy.^4,12,31,34^ In the current cases of PA-responsive degenerative myelopathy (PARDM), the lesions were characterized by the degeneration and death of neurons in the thoracic nucleus and MN of nucleus IX in the spinal cord. Under light microscopy of hematoxylin and eosin–stained preparations, neurons were pale and eosinophilic with nuclei located at the periphery of the cell body or absent and loss of Nissl granulation, which was confirmed by Toluidine blue staining. This process of neuronal degeneration is known as chromatolysis, a term applied that is broadly used to describe neuronal changes into several conditions in animals including neurodegenerative, toxic, and metabolic diseases; viral infections; and axonal injury.^34^ In our study, electron microscopy revealed changes in chromatolytic neurons similar to those observed in neurons after axonal injury rather than in hypoglycemia or excitotoxicity.^1,4,12,24^ Chromatolysis after axonal injury is due to a disruption of the protein synthesis as a result of the action of several ribonucleases.^24^ Changes involve degradation of stacks of rough endoplasmic reticulum leaving clear areas and disaggregation of polyribosomes and monoribosomes.^24^ Though not observed in piglets in this study, it has been reported that ribosomes and rough endoplasmic reticulum may also be degraded in autophagic vacuoles by ribophagy and reticulophagy, respectively.^24^

In our study of piglets with PARDM, neuronal chromatolysis and death were observed in the absence of axonal and myelin degeneration in the corresponding somatosensory tracts and motor nerve roots and nerves. These findings indicate that neuronal chromatolysis and death are the result of a primary insult to the cell bodies rather than a consequence of primary axonal damage.^24^ In contrast, axonal and myelin degeneration in first-order somatosensory tracts, in the absence of corresponding ganglionic neurons showing chromatolysis and death, provide evidence that axonal degeneration is a consequence of primary axonal damage.^24^ Yet, studies show that the site of neuronal degeneration is not a reliable indicator of where the initial injury occurred and that the sequences of events that follow are difficult to establish. For instance, a population of axons, synapses, and cell bodies showed different vulnerabilities and degenerated asynchronously.^4^

An important finding in piglets with PARDM was the accumulation of intermediary filaments in the perikaryon of neurons in the thoracic nuclei and MN in nucleus IX, as demonstrated by immunohistochemical evaluations of NFs. The accumulation of phosphorylated and non-phosphorylated NFs in the perikaryon of chromatolytic MN is a feature of neurodegenerative diseases in humans as well as in a variety of animals, including pigs.^3,6,18,28,34^ Aggregation of phospho-NFs is a hallmark of various neurodegenerative diseases such as Alzheimer’s, amyotrophic lateral sclerosis, and Parkinson’s disease.^3,6,13^ The mechanisms for accumulation of NFs are not completely understood. Deregulation of protein kinases, such as hyperactivation of cyclin-dependent kinase 5 due to neuronal insults like oxidative stress, amyloid-β toxicity, and glutamate toxicity, leads to the intraneuronal accumulation of hyperphosphorylated cytoskeletal proteins, aggregated phosphorylated NFs, and subsequent neuronal death.^6^ Interestingly, the accumulation of phosphorylated and non-phosphorylated NFs in neurons in the thoracic nucleus is a distinctive finding in piglets with PARDM compared with lower MN diseases described in pigs and other species.^18,23,29^

In this study, the majority of piglets with PARDM had degeneration and necrosis of neurons in the thoracic nucleus in both the thoracic and lumbar regions of the spinal cord and MN of nucleus IX in the ventral horn of the cervical, thoracic, and lumbar spinal cord segments. Furthermore, axonal and secondary myelin degeneration (Wallerian degeneration) was evident in the dorsal and ventral spinal nerve roots and dorsal funiculus. In an experimental study undertaken in pigs fed PA-deficient rations, lesions were confined to the peripheral sciatic and brachial nerves, neurons in the sensory spinal ganglia and nerve roots, and dorsal funiculus.^35^ These changes were characterized by chromatolysis of neurons and Wallerian degeneration of myelinated nerve fibers. A fundamental distinction between the previous experimental study^35^ and our report is the involvement of thoracic nucleus neurons and MN (nucleus IX in the ventral horn) in piglets with PARDM. In the earlier study, gait abnormalities were presented after 8 weeks of continued feeding with the deficient diet.^35^ In contrast to piglets with PARDM, pigs in that study were older (4–11 weeks) at the beginning of the trials.^35^ It is possible that the difference with respect to the distribution of the lesions in our study was due to the major metabolic susceptibility of suckling pigs to PA insufficiency.

As a differential diagnosis in piglets with PARDM, in the outbreaks in this study, we must consider the age group, nature, and pathogenesis of neuronal degeneration/necrosis, topography of the lesion, and affected functional system(s). Herein, diseases in pigs that are pertinent to discussion include pyridoxine (vitamin B6) deficiency, copper deficiency, MN diseases, hereditary porcine neuronal system degeneration, selenium toxicity, and α-Tocopherol deficiency. Pyridoxine and PA deficiencies are indistinguishable. Both deficiencies induce sensory ataxia due to lesions in proprioceptive pathways.^11,35^ While degeneration of the afferent axons is the initial and most prominent feature in pyridoxine deficiency, chromatolysis seems to be the first evidence of damage to the afferent neurons in PA-deficient animals.^11^ Copper deficiency has been described in newborn piglets and pigs up to 21 weeks of age. In contrast to piglets with PARDM, the lesion in animals with copper deficiency is characterized by primary axonal degeneration affecting the ventral and lateral funiculi of the thoracic and lumbar spinal cord, and occasionally in the brainstem and ventral peripheral nerve roots. Chromatolysis and necrosis of neurons in the brain, midbrain, and brainstem were not reported in the pigs.^27,34^ MN disease in pigs, which presumably has a hereditary basis, has been described in six 5-week-old Yorkshire and in six 6-week-old Hampshire pigs.^18,23^ Yorkshire pigs presented bilateral posterior ataxia and weakness that rapidly progressed to tetraplegia by 10 weeks of age. There was bilateral chromatolysis, degeneration, and neuronal loss restricted to motor nuclei in the ventral horns of the spinal cord, medulla oblongata, and midbrain. In addition, there was diffuse Wallerian degeneration in the ventral and lateral funiculi and ventral peripheral nerve roots and prominent atrophy of skeletal muscles, which was not present in piglets with PARDM. Ultrastructurally, the perikaryon and processes of affected neurons contained massive accumulations of NFs.^23^ No lesion was present in the dorsal funiculi and dorsal nerve root.^23^ The hereditary porcine neuronal system degeneration also results from a progressive degeneration of lower MNs.^29^ In contrast to the Yorkshire and Hampshire breeds,^18,23^ hereditary porcine neuronal system degeneration pig breeding colonies are characterized by vacuolation and deposition of osmiophilic lipid droplets in MNs in the spinal cord.^29^ Wallerian degeneration affects the sulcus marginalis and spinocerebellar tract. Axonal degeneration is solely observed in ventral spinal nerve roots and is accompanied by atrophy in skeletal muscles.^29^ Furthermore, cytoplasmic accumulation of phosphorylated and non-phosphorylated NFs demonstrated by immunohistochemistry is not a feature of this condition as it is in MN diseases in horses and in humans.^34^ In PARDM in the current study, affected pigs of the various farms presented a diverse genetic make-up ruling out a hereditary disease base.

Another important differential diagnosis in piglets of this age range is toxic myelopathy caused by selenium poisoning. The reported neurological signs of selenium poisoning are similar to those observed in piglets with PARDM, which are mainly characterized by proprioceptive and motor deficits. However, in selenium poisoning, clinical signs are the result of extensive areas of necrosis in the ventral horn (focal symmetrical poliomyelomalacia) of the cervical and lumbar intumescences.^15,30^

Lesions described in piglets with PARDM resemble a concurrent onset of neuroaxonal dystrophy/degenerative myeloencephalopathy (NAD/EDM) and equine motor neuron disease (EMND) that were recently reported in 3 young horses.^9^ The histologic lesion associated with NAD/EDM is central axonal degeneration, which is most pronounced in the somatosensory tracts (spinocuneocerebellar and dorsal spinocerebellar tract).^8,9^ Lesions associated with EMND include chromatolysis of lower MNs with perikaryal accumulation of NFs as well as peripheral axonal degeneration and associated neurogenic atrophy of muscle fibers.^9^ We were not able to measure the levels of vitamin E in piglets with PARDM. Conditions associated with temporal α-Tocopherol deficiency, such as NAD/EDM or EMND, have not yet been described in pigs, even though they have been reported in several other animal species.^22,34^

Despite the fact that pigs with PARDM presented clinical signs similar to those of previous studies,^11,34,35^ the topographic distribution of the affected neurons points to fundamental differences. Neurological signs in piglets with PARDM cannot be explained by lesions in somatosensory pathways alone, as prominent lesions also affect MNs of the ventral horn of the spinal cord. While neurological deficits due to lesions affecting MNs can be explained in a more direct manner, neurological impairments due to lesions affecting somatosensory nuclei and pathways are more complex and, as a result, more difficult to elucidate.^2,5^

The somatosensory areas affected in piglets with PARDM were the dorsal nerve rootlets, dorsal root entry zone, dorsal funiculus (dorsal column), and thoracic nucleus, which are related to proprioceptive function (Supplemental Table 2). Lesions affecting the dorsal funiculus of the cervical, thoracic, and lumbar spinal cord segments were present in 6 of 16 piglets. In comparison, lesions affecting the thoracic nucleus were present in 15 of 16 piglets. Based on the affected pathways and nucleus in piglets with PARDM, it can be suggested that unconscious rather than conscious proprioception is the primary impairment.^2,5^ Unconscious proprioceptive information to the cerebellum is conveyed by the spinocerebellar tract and cuneocerebellar tract (Supplemental Table 2). In contrast, conscious proprioceptive information is transmitted by the dorsal column-medial lemniscus system to the somatosensory telencephalic cortex (Supplemental Table 2).^2,5^

In piglets with PARDM, axonal degeneration in the dorsal nerve rootlets, dorsal root entry zone, dorsal funiculus of the posterior midthoracic and lumbar spinal cord, and thoracic nucleus (second-order neuron) suggests that both the first-order (in 7 of 16 pigs) and second-order neurons (in all 16 pigs) of these segments are primarily impaired, and that the unconscious proprioceptive information conveyed from the hind limbs is markedly disrupted.^2,5^ The presence of axonal degeneration in the dorsal nerve rootlets, dorsal root entry zone, and dorsal funiculus of the cervical spinal cord segment and the absence of lesions in the lateral cuneate nucleus (second-order neuron) indicate that the first-order neurons (spinal ganglia neurons) are primarily impaired disrupting the unconscious proprioceptive information conveyed from the front limbs.^2,5^ On the other hand, the presence of axonal degeneration in the dorsal nerve rootlets, dorsal root entry zone, and dorsal funiculus of the cervical spinal cord segment and the absence of lesions in the nerve ganglia (first-order neuron) and gracile and cuneate nuclei (second-order neurons) suggest that the axons of first-order neurons are affected and impair conscious proprioception.^2,5^

The CNS must constantly be apprised by the position, tone, and movements of the limbs and trunk. This is accomplished by proprioception input integration (primarily in the cerebellum) and by the transmission of these data back to the MNs.^2,5^ The clinical signs manifested in diseases impairing proprioceptive pathways are known as proprioceptive ataxia, which is a failure to transmit sensory information essential for smooth, coordinated motor activity, despite the motor pathway and cerebellum being intact.^2,5^ In piglets with PARDM, the clinical signs were knuckling over, hypermetria, goose stepping gait, and incoordination, which is consistent with proprioceptive ataxia. These neurological signs were more severe in the hind limbs, which coincided with a more severe and widespread lesion in the second-order neurons of the spinocerebellar tract.

In our study, pigs were not neurologically examined. However, on-site observation of animals and analysis of videos allowed us to conclude that impairment of the reflex activity that controls posture, voluntary movements, and locomotion^2,5^ is likely a consequence of degeneration and necrosis in the α-MN of the cervical and lumbar enlargements in piglets with PARDM. Eleven of 16 piglets presented lesions affecting the final common pathway for motor control and consequentially inducing lower MN syndrome. Neurological signs presented in lower MN syndrome are loss of muscle tone (weakness or paresis, flaccid paralysis), loss of muscle stretch reflexes (hyporeflexia, areflexia), muscle atrophy, fibrillation, and fasciculation.^2,5^ The lack of or low expression of ACh in affected MN, as demonstrated by immunohistochemistry with the enzyme choline acetyltransferase, supports this hypothesis. Decreased expression of neuronal ACh has been found in humans with PA deficiency.^14,21^ Furthermore, recent studies have demonstrated that cholinergic neurons are more vulnerable to deficits in acetyl-CoA than non-acetyl-CoA in chronic neurodegenerative diseases in humans.^20,41^

Because both sensory pathway inputs and α-MN are involved in the normal functioning of motor units, damage to either system will affect reflex activity that controls posture and voluntary movements, and the contribution of either to the neurological deficit in pigs with PARDM under the current study’s circumstances is difficult to determine.

In conclusion, the neurological signs in the current study were attributed to PA-responsive degenerative spinal cord disorders. All the affected piglets developed sensory ataxia and paresis. Lesions were characterized by necrosis of neurons in the thoracic nucleus and α-MN of nucleus IX of the spinal cord. Axonal degeneration was present in the roots of the dorsal and ventral spinal nerves and dorsal funiculus. Histological examination of spinal cord enlargements (C5–C7 and L1–L6), nerve roots, and thoracic segments is essential for the diagnosis of PARDM in piglets. The distribution of the lesions indicated that conscious and unconscious proprioception and the motor final common pathway were primarily affected. This study highlights the importance and practical use of detailed neuropathological analysis to refine differential diagnosis. To the best of our knowledge, there are no previous reports of degenerative myelopathy responsive to PA in suckling piglets.

## Supplemental Material

sj-pdf-1-vet-10.1177_03009858221128920 – Supplemental material for Motor and somatosensory degenerative myelopathy responsive to pantothenic acid in piglets Click here for additional data file. Supplemental material, sj-pdf-1-vet-10.1177_03009858221128920 for Motor and somatosensory degenerative myelopathy responsive to pantothenic acid in piglets by Marina P. Lorenzett, Aníbal G. Armién, Luan C. Henker, Claiton I. Schwertz, Raquel A. S. Cruz, Welden Panziera, Claudio S. L. de Barros, David Driemeier and Saulo P. Pavarini in Veterinary Pathology
