# Supplementary material for: Motor and somatosensory degenerative myelopathy responsive to pantothenic acid in piglets
Source: Vet Pathol. 2022 Oct 17;60(1):101–14. doi: 10.1177/03009858221128920 (PMC9827486; doi:10.1177/03009858221128920)
Supplement: sj-pdf-1-vet-10.1177_03009858221128920 – Supplemental material for Motor and somatosensory degenerative myelopathy responsive to pantothenic acid in piglets [file sj-pdf-1-vet-10.1177_03009858221128920.pdf]

Supplemental Materials

Motor and Somatosensory Degenerative Myelopathy Responsive to Pantothenic Acid in Piglets

Marina P. Lorenzett; Aníbal G. Armién; Luan C. Henker; Claiton I. Schwertz; Raquel A. S. Cruz; Welden Panziera; Claudio S. L. de Barros; David Driemeier; Saulo P. Pavarini.

Supplemental Figure S1. Pantothenic acid responsive degenerative myelopathy, pig, case 08. The piglet supported the body weight on the tarsus-metatarsus region, presenting light to marked "hock weight-bearing" and "hock walking".

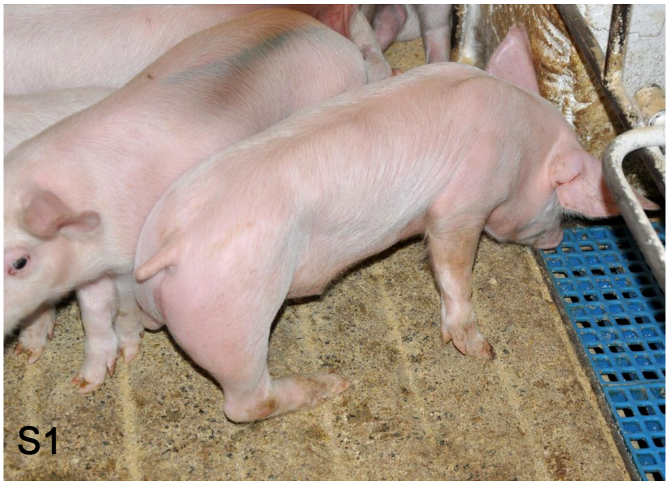

Supplemental Figure S2 - S3. Pantothenic acid responsive degenerative myelopathy, pig, case 15. Glial fibrillary acidic protein (GFAP) and ionized calcium-binding adaptor molecule 1 (Iba1) immunohistochemistry. Figure S2. Glial response in the thoracic nucleus with marked necrosis of neurons. Astrocyte reaction is demonstrated by GFAP immunohistochemistry. Figure S3. Iba1 highlighted that the predominant glial cell population was represented by microglial cells (arrow).

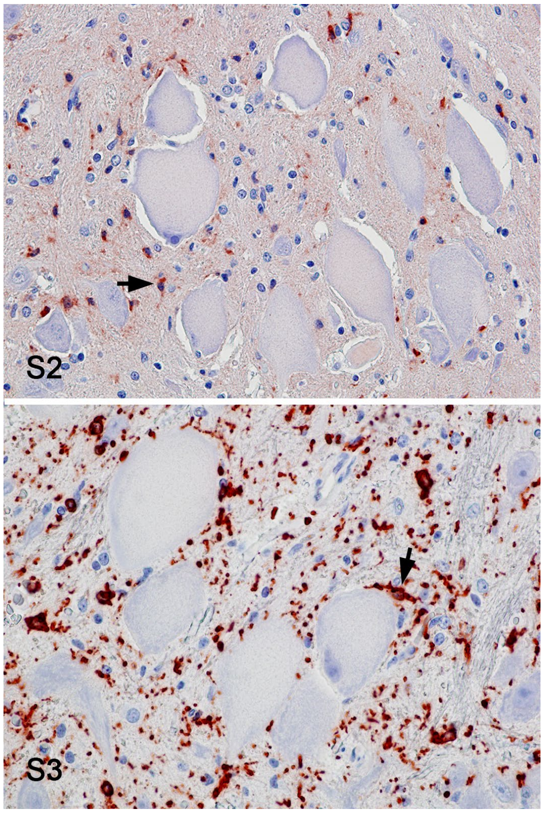

Supplemental Figure S4 - S5. Pantothenic acid responsive degenerative myelopathy, L5 spinal cord segment, pig, case 15. Toluidine blue and transmission electron microscopy. Figure S4. Cross sections of axons in the gracile fasciculus of the dorsal funiculus; a distended myelin sheath containing a gitter cell (arrow); a preserved axon (arrowhead). Figure S5. Approximate cross section of a degenerate axon (black asterisk) surrounded by myelin sheath (white arrow) that shows focal de-compacted myelin (arrowhead). The axon showed swelling and axoplasmic vesicles (V) and myelin fragments (my). Within the distended myelinated axon, there is a macrophage displaying nuclear pyknosis, (N) and phagolysosomes (SL). Adjacent, a normal axon (Ax) is highlighted for comparison.

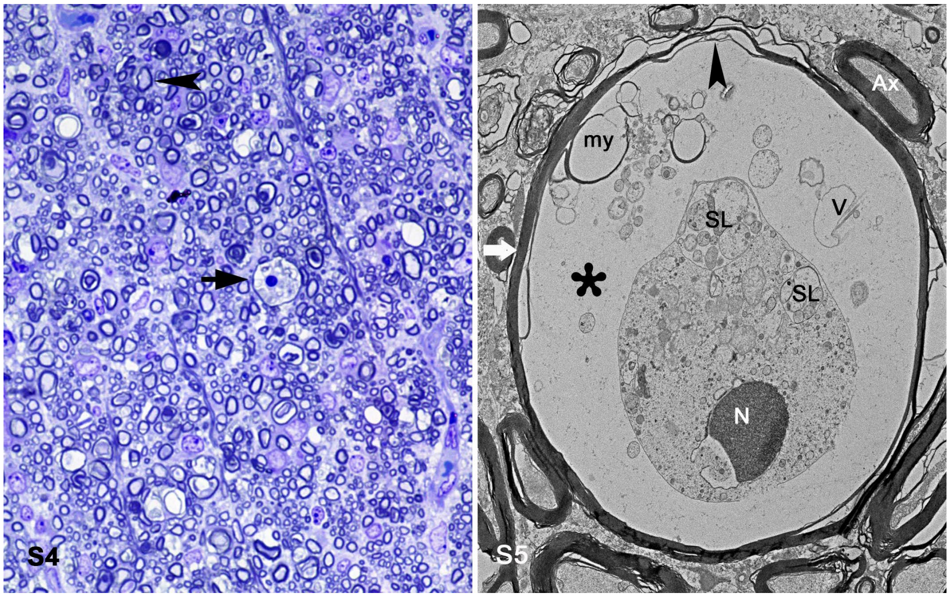

Supplemental Video 1 and 2. Pantothenic acid-responsive degenerative myelopathy, sensory ataxia, and paresis, pig 6.

Supplemental Video 3. Pantothenic acid-responsive degenerative myelopathy, proprioceptive deficit, tetraparesis, pig 8.

Supplemental Table 1: Antibody Specificity, Dilution and Isotype

Antibody Specificity	Source	Clone<br><br>Isotype	Dilution<br><br>Primary antibody<br><br>(working Ig conc.)	Incubation time/temp	Secondary antibody	Secondary antibody source
GFAP	AbDSerotec/<br>MCA4733	1B4/<br>IgG2b	1:3200 mouse -anti-GFAP (0.3ug/ml)	30 min; RT	EnVision+ goat anti-mouse	Dako
Non-phosphorylated NF	Abcam/<br>ab28029	SMI-32/<br>IgG1	1:500 mouse -anti-NF Nonphos (no conc. Data)	Overnight;<br>4° C	EnVision+<br>goat anti-mouse	Dako
CR	Abcam/<br>ab16694	IgG	1:50 rabbit-anti-Calretrine (no conc. Data)	Overnight;<br>4° C	EnVision+<br>goat anti-rabbit	Dako
NF protein	Dako/<br>M0762	2F11<br>IgG	1:2000 mouse -anti-NF (0.16μg/ml)	30 min; RT	EnVision+<br>goat anti-mouse	Dako
Iba1	Biocare/<br>CP 290A	IgG	1:400 rabbit-anti-(no conc. Data)	45 min; RT	EnVision+<br>goat anti-rabbit	Dako
ChAT	Millipore/<br>AB144P	IgG	1:1000 goat-anti-ChAT (no conc. Data)	Overnight;<br>4° C	ImmPRESS+<br>horse anti-goat	Vector

IgG = immunoglobulin G; working Ig conc. = concentration of the working dilution of immunoglobulin G; GFAP = Glial fibrillary acidic protein, NF = Neurofilament, CR = Calretinin, RT= Room temperature, Iba1 = ionized calcium binding adaptor molecule 1, ChAT = Choline Acetyltransferase, Dako = Dako Agilent Pathology Solutions, Carpinteria, California CA 93013, US; Vector = Vector Laboratories, Burlingame, California CA 94010, US.

Supplemental Table 2. Anatomic unit, pathway and function of the somatosensory and motor systems

System	Pathway/ Anatomical unit	Description	Function
Somatosensory	Spinocerebellar tract	First-order neurons of the spinocerebellar tract are located in the dorsal root ganglia. The dorsal nerve root enters the dorsolateral aspect of the spinal cord and splits into smaller rootlets. Large myelinated axons of first-order neurons, conveying information from the skin mechanoreceptors and proprioceptors, form medial bundles that enter the dorsal column. The spinocerebellar tract is further divided into dorsal and ventral segments. The dorsal spinocerebellar tract conveys ipsilateral axons from second-order neurons localized in the thoracic nucleus which is found between spinal cord segments T1 and L3 in most animal species. These axons ascend through the dorsolateral region of the lateral funiculus to the cerebellum through the caudal cerebellar peduncle.[1, 2, 3] The ventral spinocerebellar tract conveys contralateral axons from second-order neurons localized in the intermediary gray matter in the cervical and lumbar segments. These second-order neurons receive simultaneous ascending and descending information that affects motor neurons (MNs) and interneurons. Second-order axons transmit information from the front and hind limbs ascending through the ventrolateral region of the lateral funiculus to the cerebellum through the rostral cerebellar peduncle.[1, 2]	The spinocerebellar and cuneocerebellar tracts convey proprioceptive information about the activity of the effector muscles or motor neuron pools to the cerebellum. This unconscious proprioceptive information is critical for the maintenance of station and gait. Both spinocerebellar and cuneocerebellar tracts neurons act under the competitive influence of inhibitory and excitatory inputs to the spinal α-MNs and interneurons.[1]

	Cuneo-cerebellar tract	The first-order neurons of the cuneocerebellar tract are located in the dorsal root ganglia. Large first-order myelinated axons convey information from the skin mechanoreceptors and proprioceptors located in the front limbs. First-order axons form synapses with second-order neurons in the lateral cuneate nuclei located in the posterior brainstem via the cuneate fasciculus in the dorsal funiculus (dorsal column). The second-order axons from the lateral cuneate nuclei ascend in the ipsilateral caudal cerebellar peduncle.[1, 2]	
	Dorsal column-medial lemniscus system	Similar to the tracts related to unconscious proprioception pathways, first-order neurons of the dorsal column-medial lemniscal system are located in the dorsal root ganglia. The large myelinated axons of first-order neurons located in the spinal ganglia form medial bundles that enter the dorsal column. At the cervical and anterior midthoracic levels, the dorsal column consists of a medially located gracile fasciculus, which conveys input from the hind limbs to the gracile nucleus, and the cuneate fasciculus, which conveys input from the front limbs to the cuneate nucleus. Posterior to the midthoracic level, the dorsal column consists of only the fasciculus gracile. The second-order axon from the gracile and cuneate nuclei decussates in the medulla and ascends to the contralateral lateral medial lemniscus, forming the dorsal column-medial lemniscus system.[1]	The dorsal column-medial lemniscus system is critical for conscious proprioception associated with complex motor activities, such as touch, pressure, and joint proprioception, which are controlled through low-threshold mechanoreceptors.[1]
Motor	Somatic motor neurons, also referred to as lower motor	All MNs (α- and γ-MN) contain Acetylcholine as a neurotransmitter. α-MNs and γ-MNs are found in lamina IX of the ventral horn of the cervical and lumbar enlargements and are the predominant and	The motor unit is the physiological unit for all reflexes, postures, and voluntary movements. Normal

	neurons, are the final common pathway for segmental motor control.	largest of all MN. α-MN innervates the force-generating extrafusal muscle fibers. These neurons and all innervated muscle fibers constitute one motor unit. γ-MN innervates the intrafusal muscle fiber found in the muscle spindle. Also located in lamina IX of the ventral horn, γ-MNs are small neurons and account for one-third of all MNs.[1]	movements involve the coordination of up to thousands of motor units in many muscles. The segmental motor apparatus of the spinal cord is involved in: 1) reflex activity that controls posture and voluntary movements, and 2) complex motor synergies such as locomotion. γ-MN modulates the sensitivity of the muscle spindle to stretch.[1]

1. Benarroch EE, Cutsforth-Gregory JK, Flemming KD. Mayo Clinic Medical Neurosciences: Organized by Neurologic System and Levels. Mayo Clinic Scientific Press; 2018:763.
2. de Lahunta A, Glass E. Introduction. In: Veterinary Neuroanatomy and Clinical Neurology. St. Louis, MO: Saunders, Elsevier; 2009:1-5.
3. Finno CJ, Valberg SJ, Shivers J, D'Almeida E, Armién AG. Evidence of the primary afferent tracts undergoing neurodegeneration in horses with equine degenerative myeloencephalopathy based on calretinin immunohistochemical localization. Vet Pathol. 2016; 53:77–86.
